# A coimmunization vaccine of Aβ42 ameliorates cognitive deficits without brain inflammation in an Alzheimer’s disease model

**DOI:** 10.1186/alzrt256

**Published:** 2014-05-12

**Authors:** Shuang Wang, Yang Yu, Shuang Geng, Dongmei Wang, Li Zhang, Xiaoping Xie, Bing Wu, Chaofan Li, Hanqian Xu, Xiaolin Li, Yanxin Hu, Lianfeng Zhang, Christoph Kaether, Bin Wang

**Affiliations:** 1 Key Laboratory of Medical Molecular Virology of MOH and MOE, Fudan University Shanghai Medical College, 131 Dong An Road, Shanghai 200032, China; 2 State Key Laboratory for Agro-Biotechnology and College of Biologic Sciences, China Agricultural University, 2 Yuanmingyuan West Road, Beijing 100193, China; 3 Chinese Academy of Medical Sciences & Comparative Medical Center, 5 South Panjiayuan, Beijing 100021, China; 4 Institute for Age Research, Fritz Lipmann Institute, Beutenbergstraße 11, Jena D-07745, Germany; 5 Department of Pathology, College of Veterinary Medicine, China Agricultural University, 2 Yuanmingyuan West Road, Beijing 100193, China; 6 Present address: MOH Key Laboratory of Systems Biology of Pathogens, Institute of Pathogen Biology, Chinese Academy of Medical Sciences & Peking Union Medical College, Beijing, China

## Abstract

**Introduction:**

Vaccination against amyloid-β protein (Aβ42) induces high levels of antibody, making it a promising strategy for treating Alzheimer’s disease (AD). One drawback in the past was that clinical trial approval was withheld because of speculation that the Aβ42 vaccine induces CD4^+^ T cell infiltrations into the central nervous system. To reduce T-cell activation while concomitantly maintaining high anti-Aβ42 titers is a great challenge in immunology.

**Methods:**

We aimed to demonstrate that coimmunization with Aβ42 protein and expression plasmid can be beneficial in a mouse AD model and can prevent inflammation. We immunized the AD mice with the coimmunization vaccine and assessed behavior change and Aβ42 deposition. Furthermore, to determine the safety of the coimmunization vaccine, we used an induced Aβ42-EAE model to mimic the meningoencephalitis that happened in the AN-1792 vaccine clinical phase II trial and tested whether the coimmunization vaccine could ameliorate T-cell-mediated brain inflammation.

**Results:**

The coimmunization vaccination reduced Aβ plaques and significantly ameliorated cognitive deficit while inhibiting T-cell-mediated brain inflammation and infiltration. These studies demonstrate that the coimmunization strategy that we describe in this article can ameliorate AD pathology without notable adverse effects in mice.

**Conclusions:**

A coimmunization strategy leading to the development of a safe immunotherapeutic/preventive protocol against AD in humans is warranted.

## Introduction

Alzheimer’s disease (AD) is a neurodegenerative disease characterized by progressive cognitive dysfunction, massive loss of neurons and deposition of amyloid plaques and neurofibrillary tangles [1]. The pathological accumulation of amyloid is possibly due to site-specific abnormal processing of amyloid precursor protein (APP). Investigators in previous studies have shown that deposition of amyloid-β protein (Aβ), particularly the Aβ40 and Aβ42 forms, in amyloid plaques is one of the hallmarks of the disease [2-4] and could trigger a T-cell-mediated (auto)immune reactions [5]. A large body of evidence supports the amyloid cascade hypothesis, which states that accumulation of Aβ is the initiating step for the onset of AD. Therefore, most research has centered on Aβ, and many Aβ-related therapeutic strategies have been proposed and/or developed, including immunotherapy [6-9].

After successful experiments in AD model mice [8], researchers in a phase IIa immunotherapy trial in patients with mild to moderate AD showed that about 20% of vaccinees had immunoglobulin G (IgG) responses. However, the trial had to be stopped because 6% of the vaccinated patients developed meningoencephalitis [10]. Pathology reports indicated that the cases of meningoencephalitis were severe, which suggested that vaccine-induced T-cell infiltration might be the cause [11]. This raised a critical question about how to develop a vaccine that can elicit a high level of antibody against Aβ42 antigen while preventing T-cell responses [12-16]. Several approaches to answering this question have been employed, including the use of truncated versions of Aβ42 that exclude T-cell epitopes (for example, Aβ1–28, Aβ1–16, Aβ1–14 and Aβ1–9). In many of the previously reported Aβ immunization studies, researchers have found reduced cerebral Aβ levels and/or improved cognition in mice, nonhuman primates and humans [15,17-21]. Monoclonal antibodies against Aβ42 have been used in passive vaccination [22-25], including bapineuzumab, solanezumab and ponezumab [26-28]. Although selecting the B-cell epitopes for vaccines could avoid the T-cell response, short peptides do not have the same strong ability to stimulate a high titer of IgG and would therefore need to be modified, adding to the production cost and complexity of vaccine studies in AD [29]. Passive immunization with an antibody may not stimulate the unwanted T-cell response, but antibodies are more expensive than protein or DNA vaccines and do not last very long *in vivo*. The various strategies being used for vaccine development are at different stages, and some have a bearing on the use of DNA and protein coimmunization or the induction of anti-inflammatory, regulatory T cells (Tregs). Lambracht-Washington *et al*. demonstrated a novel strategy of using peptide prime plus DNA boost immunization regimens [30]. These regimens induced higher levels of anti-Aβ42 antibody responses, mainly of T helper type 2 (Th2) cells rather than Th1 cells, in contrast to traditional Aβ42 peptide immunizations. Furthermore, T-cell responses were lower before than after peptide immunization. Although the protocol demonstrated a potential alternative way to generate high levels of antibody against Aβ plaques, T-cell responses potentially could be autoreactivated if multiple immunizations were required. Liu *et al.*[29] used a diphtheria toxoid (DT)-conjugated Aβ1–15 vaccine that deleted the T-cell epitope and found that it upregulated CD25^+^ Tregs. Whether these regulatory T cells were specific for the Aβ peptide and had a major anti-inflammatory impact needs to be further investigated. The results obtained with other vaccines have indicated a capacity to induce T cells with the CD4^+^CD25^+^Foxp3^+^ regulatory phenotype that is responsive to Aβ15-DT, but not to the Aβ1–15 epitope or to intact Aβ1–40/Aβ1–42 [29]. This finding indicates that induction of antigen-specific regulatory T cells (iTregs), even when not directly against an Aβ antigen, may improve the safety of Aβ-based vaccines against inflammation.

Tregs have been shown to be important regulators of tolerance, which suggests the possibility of developing direct strategies to use these cells to prevent or even to treat autoimmune inflammation [31]. Various approaches to inducing Treg cells to constrain autoreactive T cells have been explored. Notably, the induction of antigen-specific iTreg cells targeted to allergy, asthma and autoimmune disease antigens is considered a promising immunomodulatory strategy [32-34]. Therefore, it may be possible to develop a strategy to induce antigen-specific iTreg cells targeted against Aβ42 antigen without affecting the antibody response.

We have previously reported that coimmunization with a DNA vaccine and its encoded protein antigen induced iTreg to inhibit antigen-specific T-cell function *in vivo*. Importantly, this suppression had no effect on antibody production [33]. In this article, we demonstrate that coimmunization with Aβ42 protein and a DNA vaccine encoding Aβ42 in an AD mouse model induced high titers of anti-Aβ42 antibodies, which resulted in clearance of amyloid plaques and improvement in cognitive behavior. Strikingly, the coimmunization also induced antigen-specific iTreg cells that ameliorated T-cell-mediated brain inflammation. We show that the prevention of inflammation is due, at least in part, to the suppressive effects of iTregs on CD4^+^ effector T-cell (Teff) function.

## Methods

### Animals

Adult female C57BL/6 mice (6 to 8 weeks of age) were purchased from the Animal Institute of the Chinese Medical Academy (Beijing, China). APP_695_ mice (10-month-old, C57BL/6 background) [35-37] were obtained from the Chinese Academy of Medical Sciences & Comparative Medical Center. Mice that coexpress Foxp3 and enhanced green eGFP (Foxp3-eGFP, C57BL/6 background) were kindly provided by Dr Minghui Zhang (Tsinghua University, Beijing, China). APP/PS1 mice (C57BL/6 background) [38] were kindly provided by Mathias Jucker, Hertie Institute, Tübingen, Germany. All mouse protocols were approved by the animal welfare committees of China Agricultural University, Fudan University and the Institute for Age Research. All animals were maintained under specific pathogen-free conditions and a 12-hour light–dark cycle.

### Reagents

Recombinant eukaryotic and prokaryotic plasmids were identified by restriction enzyme digestion and verified by sequencing analysis. The inserts were subcloned into either a pVAX1 or pET28a vector (Invitrogen, Carlsbad, CA, USA). Expression levels were examined by Western blot analysis 48 hours after transfection of BHK cells (American Type Culture Collection, Manassas, VA, USA) for inserts in the pVAX backbone or 8 hours after induction of transfected *Escherichia coli* with 0.1 mM isopropyl-β-D-thiogalactopyranoside for inserts in pET28 vectors.

### Immunization

Female C57BL/6 mice (6 to 8 weeks old) and APP_695_ mice (both male and female, 10 months old) were immunized with various regimens via the tibialis anterior muscle on days 0, 14, 28 and 70. These regimens were as follows: Aβ42 protein, 200 μg/mouse; pVAX1-Aβ42, 100 μg/mouse; coimmunization, a mixture of 200 μg of Aβ42 protein and 100 μg of pVAX1-Aβ42; a positive control in which mice were first immunized with 200 μg of Aβ42 protein emulsified with complete Freund’s adjuvant (CFA; Sigma-Aldrich, St Louis, MO, USA), and then second, third, fourth and fifth immunizations were delivered with 200 μg of Aβ42 protein in incomplete Freund’s adjuvant (IFA; Sigma-Aldrich).

### Flow cytometry

T cells were isolated from the spleens of immunized C57BL/6 mice or APP_695_ transgenic mice on day 7 after the fourth immunization. For intracellular staining, T cells were stimulated with Aβ42 protein at 10 μg/ml for 8 hours and subsequently treated with brefeldin A (BFA; BD Biosciences, San Diego, CA, USA) for 2 hours *in vitro*. The cells were blocked with BD Fc Block antibody (BD Biosciences) in phosphate-buffered saline (PBS) for 30 minutes at 4°C before being fixed with 4% paraformaldehyde and permeabilized with saponin (Sigma-Aldrich). The splenocytes were intracellularly stained with the appropriate concentrations of phycoerythrin (PE)-labeled antibodies, including anti-Foxp3, interleukin 10 (anti-IL-10), transforming growth factor β (anti-TGF-β), interferon γ (anti-IFN-γ), anti-IL-4 and PE-cyanine 5.5 (PE-Cy5.5)-labeled anti-CD25 antibodies (eBioscience, San Diego, CA, USA) and fluorescein isothiocyanate (FITC)-labeled anti-CD4 antibody (eBioscience) for 30 minutes at 4°C. The cells were washed and analyzed with a FACSCalibur flow cytometer equipped with CellQuest Pro software (BD Biosciences).

### Immunofluorescence staining

For Aβ plaque immunohistochemistry, frozen brain sections taken from 9-month-old APP/PS1-transgenic mice were warmed to room temperature for 30 minutes and fixed in ice-cold acetone for 5 minutes before being air-dried for 30 minutes. The sections were incubated with normal serum for 30 minutes to block nonspecific binding of immunoglobulin. The tissue sections were reacted first for 1 hour at room temperature or overnight at 4°C with (1) antisera induced by coimmunization or by protein vaccination, or (2) with sera from untreated mice (all sera were at 1:200 dilution) or (3) with monoclonal antibody 3552 (1:2,000 dilution) as a positive control. All serum dilutions were made in primary antibody dilution buffer. After incubation, the sections were washed three times with PBS, then incubated with Alexa Fluor 555–labeled secondary antibody (1:1,000 dilution; Invitrogen) for 30 minutes at room temperature and washed three times with PBS. After brush transfer of sections onto glass slides, plaques were detected by fluorescence microscopy (Carl Zeiss, Jena, Germany) at 546-nm emission. The nuclei were stained with 4′,6-diamidino-2-phenylindole (DAPI), and the sections were incubated with 1 μg/ml DAPI for 5 minutes in the dark at room temperature, washed twice with PBS and detected at 405-nm emission.

### T-cell proliferative response

Single-lymphocyte suspensions were obtained from the spleens of immunized C57BL/6 mice or APP_695_ mice on day 7 after the fourth immunization. Cells in RPMI 1640/Ham’s F-10 Nutrient Mix medium (GIBCO, Eggenstein, Germany) containing 10% fetal bovine serum were used to determine the T-cell proliferative response by 3-(4,5-dimethylthiazol-2-yl)-2,5-diphenyltetrazolium bromide (MTT) staining after Aβ42 stimulation *in vitro* for 72 hours. MTT-stained cells were analyzed using an enzyme-linked immunosorbent assay (ELISA) plate reader (Magellan; Tecan Austria, Grodig, Austria) at 450-nm absorbance. The data are expressed in SI units, and the means are the product of (stimulated cells – medium) ÷ (unstimulated cells –medium) detected at 450-nm absorbance.

### Measurement of Aβ42 protein-specific antibodies

The level of anti-Aβ42 serum IgG antibodies was determined by ELISA in 96-well plates coated with Aβ42 protein at 10 μg/ml and detected with a secondary goat anti-mouse IgG antibody conjugated with horseradish peroxidase (HRP; Bio-Rad Laboratories, Hercules, CA, USA). Absorbance at 450 nm was measured with a Magellan ELISA plate reader.

### Fibrillar Aβ deposition and histological analysis

Thioflavin S staining was used, which is common for fluorescent staining of fibrillar amyloid deposition [39]. Brain tissue was fixed in 4% neutral buffered formalin and mounted in paraffin blocks. After deparaffinization and hydration, the sections were washed in PBS and incubated in a solution containing 0.25% potassium permanganate and 1% oxalic acid until they appeared white. The sections were then washed in water and stained for 3 minutes with a solution of 0.015% thioflavin S in 50% ethanol. Finally, the sections were washed in 50% ethanol and in water, then dried and dipped in Histo-Clear (National Diagnostics, Atlanta, GA, USA) histological clearing agent before being coverslipped with Permount (Sinopharm Chemical Reagent Co., Ltd, Shanghai, China) mounting medium [40]. The sections were examined using three equidistant sections per animal at 488-nm emission. Quantification and statistical analysis of staining were carried out using Image-Pro Plus version 6.0 software (Media Cybernetics, Rockville, MD, USA).

### Immunohistochemical staining

For Aβ plaque immunohistochemistry, brain tissue was fixed in 4% neutral buffered formalin and mounted in paraffin blocks. After deparaffinization and hydration, tissue sections in citric acid buffer solution (pH 6.0, antigen repair) were placed in the microwave for antigen retrieval. Next, sections were put into 3% hydrogen peroxide solution, incubated at room temperature for 25 minutes and placed in the PBS solution (pH 7.4) and then shaken and washed for 5 minutes each time to block endogenous peroxidase. The tissue sections were reacted first for 1 hour at room temperature or overnight at 4°C with monoclonal antibody anti-Aβ (1:200 dilution; Santa Cruz Biotechnology, Santa Cruz, CA, USA) or anti-CD4 (1:100 dilution; ABclonal, Wuhan, China). All primary antibodies were diluted in 5% bovine serum albumin (BSA)/PBS buffer and incubated with the sections before they were washed three times with PBS and then incubated with HRP-labeled secondary antibody (1:200 dilution; KPL, Gaithersburg, MD, USA) for 50 minutes at room temperature. 3,3′-Diaminobenzidine coloration (Invitrogen) was carried out, and the reaction product was kept at room temperature without light for 10 minutes and stained with hematoxylin and eosin (H&E). All of the sections were detected with an EVOS FL cell imaging system (Life Technologies, Carlsbad, CA, USA) in visible light. Quantification and statistical analysis of staining were carried out using Image-Pro Plus version 6.0 software (Media Cybernetics).

### Microhemorrhage staining and hematoxylin and eosin staining

For microhemorrhage staining, brain tissue taken from 14-month-old AD mice after the fifth immunization was mounted and stained for hemosiderin using 2% potassium ferrocyanide in 2% hydrochloric acid for 15 minutes, followed by counterstaining in a 1% neutral red solution for 10 minutes at room temperature. Microhemorrhage events in the form of Prussian blue-positive profiles were observed on all brain tissue sections from each mouse by microscopy (Life Technologies) in visible light. For H&E staining, the brains taken from the Aβ42 autoimmune encephalomyelitis (Aβ42-EAE) mice on day 21 after induction were fixed in 4% paraformaldehyde. Paraffin sections were stained with H&E and observed by microscopy (Life Technologies) in visible light.

### Plasmid expression tests

The BHK-21 cell line was used for detecting pVAX1-Aβ42 transfection and expression with Lipofectamine 2000 reagent (Invitrogen). The BHK-21 cells were put into 24-well plates at 10^5^ per well. The plasmid was transfected using 1 μg of plasmid and 2 μl of Lipofectamine 2000 in 500 μl of serum-free medium. Serum-free medium was replaced after 3 hours with serum-containing medium. The whole operation was carried out according to the Lipofectamine 2000 kit instructions. The cells were collected in radioimmunoprecipitation assay lysis buffer (Beyotime, Shanghai, China) at 48 hours after transfection, and the plasmid expression was tested by Western blot analysis. The primary anti-Aβ42 antibody (1:1,000 dilution; Santa Cruz Biotechnology) or glyceraldehyde 3-phosphate dehydrogenase antibody (anti-GAPDH; Abcam, Cambridge, MA, USA) was incubated overnight at 4°C and washed. The HRP-labeled anti-mouse IgG secondary antibodies (1:3,000 dilution; Santa Cruz Biotechnology) were incubated for 1 hour at room temperature and washed. The blots were analyzed by chemiluminescence detection.

### Behavioral tests

#### Open-field test

Behaviors of APP_695_ mice were measured using the open-field test. We used EthoVision XT monitoring and analysis software (EthoVision XT; Noldus Ltd, Wageningen, the Netherlands) and a 50 × 50–cm open field. The open field was divided into three areas, which included the peripheral zone (zone 1) and the central zone (zones 2 and 3). Zone 1 was 8 cm away from the edge of the open field. Zone 3 occupied the central area, which was 16% of the total. The remaining area was designated zone 2. Each mouse had 5 minutes of free movement in the open field. We recorded the length of time that each mouse stayed in each zone and the frequency that the mouse was in the mobile state. Mobility was the state variable and included three different variables: immobile, mobile and highly mobile. The mouse was considered to be immobile when the change in area of the mouse between current sample and the previous sample (referred to as the *changed area*) was <20%, highly mobile when the changed area was >60% and mobile when the changed area was between 20% and 60%. The amount of time spent in the peripheral zone is a manifestation of thigmotaxis [41]. Room temperature was kept constant, and the light level was even across the open field. The open field was wiped clean with 75% alcohol and dried before each experiment to remove residual odors.

#### Morris water maze

Learning and memory were tested using the Morris water maze (MWM) 1 day after the end of the open-field test. The protocol for the MWM test was modified from previously reported methods [42,43]. Briefly, the apparatus included a pool with a diameter of 100 cm that was filled with opaque water at 20°C ± 1. An escape platform 15 cm in diameter was placed 0.5 cm below the water surface. Geometric objects with contrasting colors were set at the remote ends of the water tank as reference points. Room temperature was kept constant at 20°C ± 1, and the light level was even throughout the room. Spatial memory was assessed by recording the latency time for the animal to escape from the water onto a submerged escape platform during the learning phase. The platform was in zone 4 and defined as zone 5. The mice were studied in four trials per day for 3 consecutive days. The mice were allowed to stay on the platform for 15 seconds before and after each trial. The time that it took for an animal to reach the platform (the latency period) was recorded. Twenty-four hours after the learning phase, the mice swam freely in the water tank without the platform for sixty seconds, and the time spent in the region was recorded. Monitoring was performed with a video tracking system (EthoVision XT).

### Detection of insoluble Aβ42 by enzyme-linked immunosorbent assay

The brains of APP_695_ mice were extracted in Tris-HCl buffer at pH 8.0 and centrifuged at 4°C and 20,000 × *g* for 20 minutes. After we discarded the supernatant, we washed the pellet in Tris-HCl pH 8.0 buffer three times and homogenized it in buffer composed of 6 M guanidine hydrochloride with -Tris-HCl pH 8.0. After it was sonicated at room temperature for 30 seconds, the sample was incubated for 1 hour at room temperature and then centrifuged at 4°C and 20,000 × *g* for 20 minutes. Samples were diluted 1:12 in ELISA bicarbonate buffer pH 9.6, incubated in 96-well plates at 4°C overnight and assayed with a primary anti-Aβ antibody (2B9, diluted 1:500; Santa Cruz Biotechnology) and secondary goat anti-mouse IgG antibody conjugated with HRP (diluted 1:1,000; Bio-Rad Laboratories). Absorbance at 450 nm was measured with an ELISA plate reader (Magellan).

### Analysis of inflammatory cytokines

Brain tissue was homogenized by ultrasonication in cold PBS after perfusion. After supernatants were centrifuged at 8,000 rpm/min at 4°C for 30 minutes, they were diluted 1:10 with cold PBS and assayed by cytometric beads array (CBA; BD Biosciences) so that we could detect expression levels of IL-6, IL-1β, tumor necrosis factor α (TNF-α) and IFN-γ.

### Induction of experimental autoimmune encephalomyelitis

We used a myelin oligodendrocyte glycoprotein–induced EAE (MOG-EAE) model. Female 8-week-old C57BL/6 mice were immunized with 200 μg/100 μl MOG 35–55 peptide fragment emulsified in 100 μl of 5× CFA (500 μg/100 μl killed H_37_Ra/CFA; Sigma-Aldrich). We injected a total of 200 μl of CFA into the animals, which was divided between two subcutaneous posterior auricular sites. Pertussis toxin (PT) (200 ng/mouse; Sigma-Aldrich) was injected intraperitoneally on days 0 and 1 [44].

#### Aβ42-EAE-induced brain inflammation

To mimic the results obtained from AN-1792 vaccine-induced brain inflammation and encephalitis [11], we used Aβ42 protein (200 μg/100 μl) to replace MOG in the MOG-EAE model described in the preceding subsection and used this Aβ42-EAE model as the positive experimental control. After EAE induction, the mice were immunized using various regimens via the tibialis anterior muscle on days 0 and 14. The regimens used were as follows: the protein group was immunized with Aβ42 protein at 200 μg/mouse, the plasmid group was immunized with pVAX1-Aβ42 at 100 μg/mouse and the coimmunization group was immunized with a mixture of 200 μg of Aβ42 protein and 100 μg of pVAX1-Aβ42. We used the untreated MOG-EAE or Aβ42-EAE model mice as positive controls. On day 21, the mice were anesthetized before heart perfusion with cold PBS. The brain tissues were isolated and digested with trypsin (MACGENE, Beijing, China) at 37°C for 0.5 hour to obtain single-cell suspensions. The brain cells were stained with FITC/anti-CD3 and allophycocyanin (APC)/anti-CD4 antibodies (eBioscience).

### CD25^−^ induced antigen-specific regulatory T-cell transfer assay

Foxp3-eGFP mice were used as donor mice and were coimmunized intramuscularly twice (with a 14-day interval) with a mixture of 200 μg of Aβ42 protein and 100 μg of pVAX1-Aβ42. Splenocytes were isolated from the coimmunized donor mice 7 days after the second immunization, stained with APC anti-mouse CD4^+^ and Foxp3^+^ and PE-Cy5-labeled anti-mouse CD25^+^ antibodies, and sorted using a BD FACSAria II flow cytometer. Any cells stained for CD25^+^ were excluded. The sorted CD25^−^ iTreg cells were adoptively transferred intravenously at 10^6^ per animal into C57BL/6 mouse recipients that had been immunized twice with CFA-emulsified Aβ42 protein to induce Aβ42-EAE-induced inflammation.

On day 21, all recipients were anesthetized, and heart perfusion was performed with cold PBS. The brains were separated and digested with trypsin at 37°C for 0.5 hour to obtain single-cell suspensions as described in the preceding subsection. All cells were stained with FITC/anti-CD3 and APC/anti-CD4 antibodies.

### Statistical analysis

The results are presented as means ± SD. Except for data derived from the MWM experiments, statistical analysis was carried out by parametric one-way analysis of variance (ANOVA) and *t*-tests were used for comparisons between two groups. In the MWM tests, escape latency in the hidden platform trial was analyzed using two-way ANOVA of repeated measures, and one-way ANOVA was conducted to assess the data obtained from the probe trial. Differences were considered to be statistically significant at *P* < 0.05.

## Results

### Aβ42 coimmunization induces high immunoglobulin G titer anti-Aβ42 antibodies capable of binding to amyloid plaques

**Figure 1:**
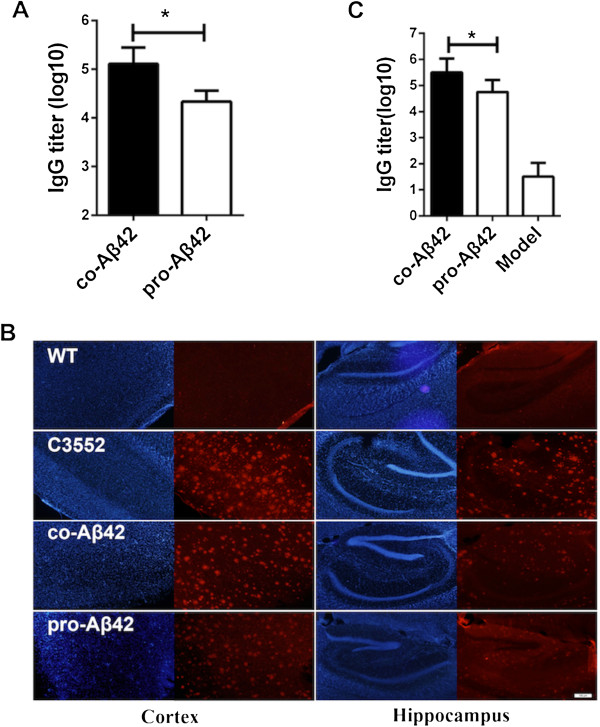
**Coimmunization against Aβ induces high levels of anti-Aβ42 immunoglobulin G.** Serum samples were collected 7 days after the last immunization of C57BL/6 mice **(A)** and amyloid precursor protein APP_695_ mice **(C),** and the anti-Aβ immunoglobulin G (IgG) titer was determined by enzyme-linked immunosorbent assay (ELISA). Mean titers (*n* = 6) are expressed in log_10_ units. **(B)** Immunofluorescent staining of hippocampal and cortical tissue sections of 9-month-old APP/PS1-transgenic mice. The sections were stained with anti-sera from untreated mice (naïve; wild type (WT)), coimmunized mice (co-Aβ42) and protein-immunized mice (pro-Aβ42). Polyclonal anti-Aβ antibody 3552 served as a positive control. Red: secondary antibody; Blue: 4′,6-diamidino-2-phenylindole. All data are presented as mean ± SD. Statistical analysis was performed using parametric one-way analysis of variance, and *t*-tests were used to compare two groups. The results of one of three independent experiments are shown. *P < 0.05.

Anti-Aβ antibodies are considered to be the main contributors to the efficacy of AD vaccines [8,27]. To test whether Aβ42 protein vaccine or Aβ42 protein plus Aβ42-coding DNA vaccine (coimmunization according to the regimen described in the Methods section) could elicit a robust anti-Aβ42 immune response, C57BL/6 mice were immunized intramuscularly at biweekly intervals. Serum samples were taken, and ELISA was performed to measure IgG titers. The coimmunized group had higher IgG titers than the protein-only vaccine group (*P* < 0.05) (Figure 1A). The IgG titers induced by the DNA vaccine were 2 log_10_ lower than in the coimmunized group and 1 log_10_ lower than in the protein vaccine group (Additional file 1: Figure S1A). To further test the specificity of the antibodies generated in immunized mice, sera were used to immunostain amyloid plaques from APP/PS1 mice. The APP/PS1 mouse is a model of AD characterized by early and massive accumulation of amyloid plaques [38]. As shown in Figure 1B, serum samples from the coimmunized group strongly reacted with plaques in the cortex and hippocampus, similar to the positive control polyclonal anti-Aβ antibody 3552. We also used another well-established model, the APP_695_ transgenic mouse. This mouse line harbors an APP_695_ transgene and, after 8 months, develops amyloid plaques and displays behavioral deficits in open-field tests and the MWM [36,37]. To assess whether coimmunization could also induce anti-Aβ42 IgG in these mice, we performed ELISA to measure the IgG titer after the fourth immunization. The result showed that anti-sera from these coimmunized mice also had higher IgG titers than those from protein-immunized mice (Figure 1C). These results demonstrate that the coimmunization strategy caused robust production of potentially plaque-clearing anti-Aβ42 antibodies.

### Coimmunization of Aβ42 reduces plaque formation in APP_695_ mice

**Figure 2:**
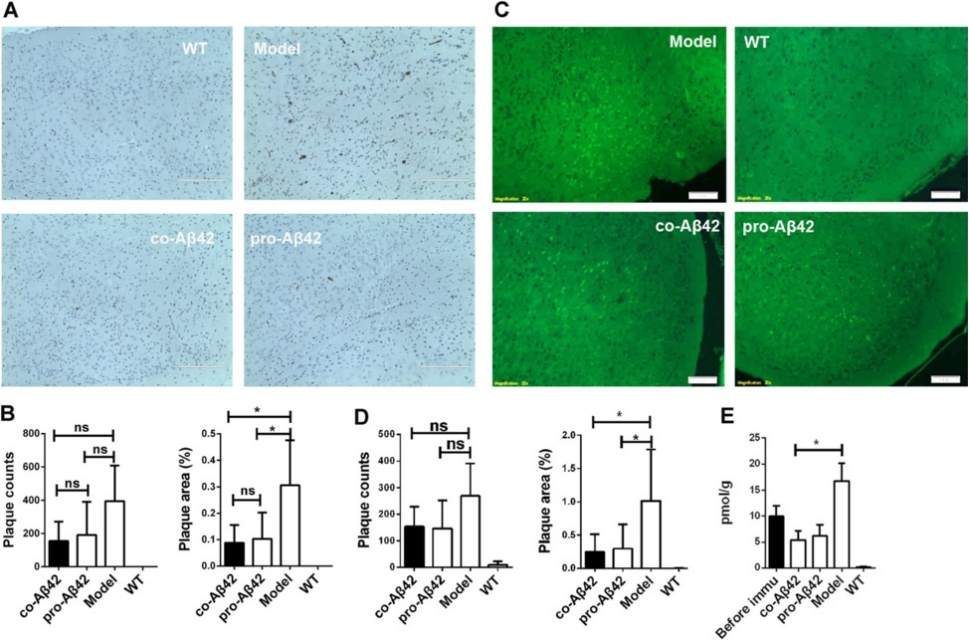
**Coimmunization reduces amyloid-β deposition in brains of amyloid precursor protein APP**_**695 **_**mice.** Brain sections were taken from 14-month-old Alzheimer’s disease (AD) mice (*n* = 5) after the fifth immunization and stained by immunohistochemistry as indicated in the Methods section. **(A)** Plaques revealed by immunohistochemistry. The sections were incubated with amyloid-β42 antibody (anti-Aβ42) used as the primary antibody. WT, wild-type littermates used as negative controls; model, amyloid precursor protein 695 (APP_695_) mice not immunized; co-Aβ42, APP_695_ mice immunized with co-Aβ42 vaccine; pro-Aβ42, APP_695_ mice immunized with protein vaccine. **(B)** Cortical sections were viewed under a microscope with visible light, and quantification and statistical analysis of plaque counts and plaque area were performed using the Image-Pro Plus version 6.0 software program. **(C)** Brain sections were taken from 14-month-old AD mice (*n* = 5) after the fifth immunization and stained with thioflavin S. **(D)** Cortical sections were viewed under the fluorescence microscope at 488-nm emission, and quantification and statistical analysis of plaque counts and plaque area were performed using the Image-Pro Plus version 6.0 software program. **(E)** The remaining brain tissues were used to isolate the insoluble protein fraction and analyzed by enzyme-linked immunosorbent assay with a specific monoclonal antibody against Aβ. All data are presented as mean ± SD. Statistical analysis was performed using parametric one-way analysis of variance, and *t*-tests were used to compare two groups. ns, Not significant. *P < 0.05 compared with APP_695_ mice.

To investigate whether the coimmunization regimen could reduce amyloid plaque formation effectively *in vivo*, APP_695_ mice were killed 14 days after the last immunization, and their brain tissue was analyzed for plaque deposition by performing either ELISA against Aβ42 antigen or fibrillar amyloid–specific thioflavin S staining. The ELISA result shows that insoluble Aβ42 deposition in brain tissue was significantly reduced in the coimmunized and protein-immunized groups compared to the untreated APP_695_ mice (*P* < 0.05) (Figure 2E).

The brain cortex sections were stained with thioflavin S, and the number of plaques as well as the total plaque area were calculated (Figures 2C and 2D). As expected, the APP_695_ model group had the highest plaque counts, and the immunized groups had lower plaque counts, albeit not significantly. However, the plaque areas were significantly smaller in both the coimmunization and protein-immunized groups than in the APP_695_ model group (Figure 2B). This finding is in good agreement with the results obtained with immunohistochemical staining (Figures 2A and 2B). The results show that the APP_695_ mice also had developed both diffused and some focused Aβ deposits and that the immunizations with vaccines could facilitate clearing of the plaques. In summary, these results indicate that both the coimmunization and protein immunization regimens effectively reduced plaque loads in APP_695_ mice.

### Coimmunization of Aβ42 attenuates abnormal exploratory activity of APP_695_ mice in open-field test

**Figure 3:**
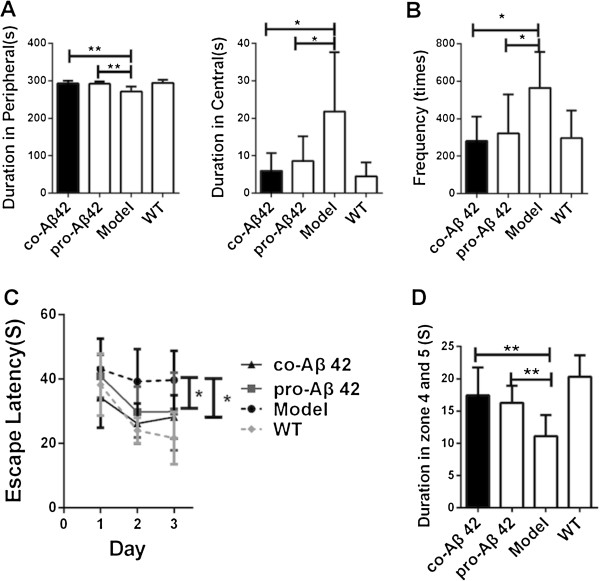
**Coimmunization improves behavior of amyloid precursor protein 695 mice in open-field and Morris water maze tests.** Groups of amyloid precursor protein 695 (APP_695_) mice (*n* = 9) were treated with the indicated vaccines. Age- and sex-matched nontransgenic littermates (that is, wild type (WT)) that did not receive any treatment were used as controls. APP_695_ mice without vaccination were used as the Alzheimer’s disease (AD) model controls. **(A)** Thigmotaxis was assessed on the basis of the amount of time spent in the peripheral and central zones with regard to autonomous behaviors in the open-field test. **(B)** Graph showing the frequency of exhibition of total mobile changes in the open-field test. **(C)** Graph of the learning and memory abilities demonstrated in the Morris water maze. Plotted is the escape latency during 3 days of hidden platform tests. **(D)** After withdrawal of the platform on the fourth day, the time spent in the original underwater platform zones 4 and 5 was determined. All data are presented as mean ± SD. For the Morris water maze tests, escape latency in the hidden platform trial was assessed by two-way analysis of variance (ANOVA) of repeated measurements. One-way ANOVA was used for the data obtained from the probe trial. We used *t*-tests to compare two groups. *P < 0.05 and **P < 0.01 compared with AD model mice. co-Aβ42, APP_695_ mice immunized with co-amyloid-β42 vaccine; pro-Aβ42, APP_695_ mice immunized with amyloid-β42 protein vaccine.

The open-field test is a method of qualitatively and quantitatively measuring general locomotor activity and willingness to explore in rodents. The hamster prion promoter (hPrP)-APP transgenic mouse models display a disinhibition-like phenotype in the elevated-plus maze and hyperactivity in some areas, such as in the open field [45-47], which we confirmed for APP_695_ mice compared to wild-type (WT) mice (Figure 3A). Compared with WT mice, the APP_695_ AD mice spent more time in the central zone (zone 2 and zone 3) and less time in the peripheral zone of the field (Figure 3A) and showed an increased frequency of total mobility (*P* < 0.05) (Figure 3B). The behavior of the coimmunized and Aβ42 protein–immunized mice resembled that of their WT littermates, with more time spent in the periphery and less in the center. Likewise, the frequency of total mobility was significantly decreased compared with the untreated AD mice (Figures 3A and 3B). The DNA-immunized group also showed some improvements, but the differences were not significant compared with those in the coimmunized and Aβ42 protein–immunized groups (data was not shown).

### Coimmunization of Aβ42 improves the learning and memory of APP_695_ mice in Morris water maze tests

To assess spatial learning and memory function, all animals underwent MWM tests 14 days after their last immunization. As shown in Figure 3C, there was a significant difference overall in escape latency between the four groups (group effect: *F*(4,36) = 11.13, *P* < 0.01; training day effect: *F*(4,144) = 12.45, *P* < 0.01; and group × training day interaction: *F*(12,144) = 1.087, *P* > 0.05). The latency to find the submerged platform decreased every day in each group, but the escape latency in the untreated APP_695_ mice was significantly longer than that of the WT group (*P* < 0.01). The coimmunized and protein-immunized AD mice showed significantly decreased escape latencies compared with those of the untreated APP_695_ controls (*P* < 0.05), almost down to WT levels. The DNA-immunized mice also improved their learning behavior, but not significantly compared to the coimmunization and Aβ42 protein-immunized groups (data not shown).

In a probe test, the frequency of crossing the platform was measured for 60 seconds on the fourth day after the last acquisition test. As shown in Figure 3D, there was an overall significant difference in the frequency of crossing the platform between the five groups (*F*(4,36) = 10.45, *P* < 0.01). The latency was decreased in the untreated APP_695_ mice compared with the WT mice (*P* < 0.01). Duration times in the platform zone were significantly increased (*P* < 0.01) among both the coimmunized and protein-immunized groups compared with the untreated APP_695_ mice, whereas the DNA-immunized group showed no improvements compared with the AD mice (*P* > 0.05) (data not shown). Taken together, these results show that that Aβ42 protein immunization as well as coimmunization efficiently ameliorated cognitive deficits in AD mice.

### Coimmunization affects inflammatory cytokines in brain tissue

**Figure 4:**
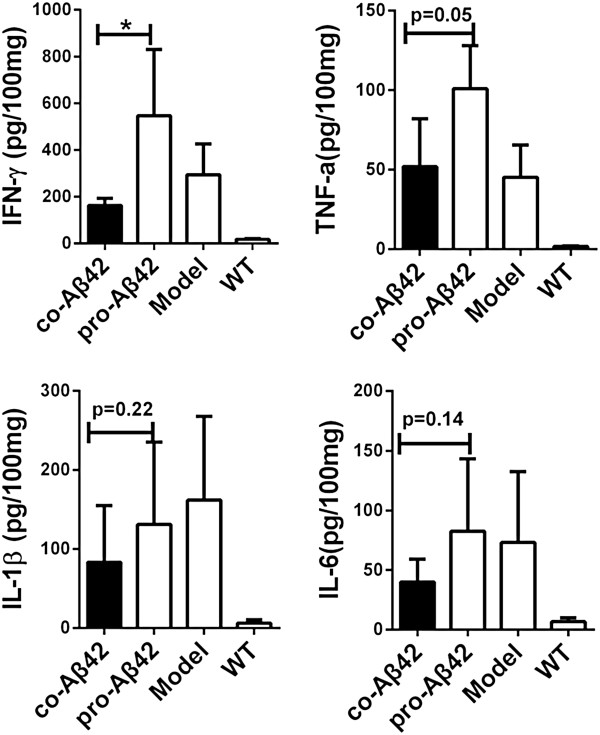
**Coimmunization suppressed the expression of inflammatory cytokines in the brains of amyloid precursor protein 695 transgenic mice.** Protein lysates were prepared from the brains of wild-type (WT), amyloid protein precursor 695 (APP_695_) and vaccinated mice 7 days after the fifth immunization (*n* = 5). The expression of cytokines was determined by cytometric bead array. Levels of interferon γ (IFN-γ), tumor necrosis factor α (TNF-α), interleukin 1β (IL-1β) and IL-6 were analyzed, and mean ± SD values are expressed as picograms per 100 milligrams of brain tissue. Statistical analysis was performed using parametric one-way analysis of variance, and *t*-tests were used to compare two groups. *P < 0.05 compared with APP_695_ mice. co-Aβ42, APP_695_ mice immunized with co-amyloid-β42 vaccine; pro-Aβ42, APP_695_ mice immunized with amyloid-β42 protein vaccine.

Inflammatory cytokines in brain tissue are believed to induce brain inflammation and, in severe cases, meningoencephalitis. To compare the level of cytokines in the brain tissue of the immunized and control groups, brain tissue was homogenized and assayed for cytokine expressions by CBA (Figure 4). The results show that there was an elevated basal inflammatory state in the brain of APP_695_ mice compared to WT mice, which is indicated by increased expression of IFN-γ, TNF-α, IL-1β and IL-6. Aβ42 protein vaccination induced further increases in IFN-γ and TNF-α levels compared to those of untreated APP_695_ mice. In contrast, in the coimmunization group, cytokine levels were similar to those of untreated APP_695_ mice (TNF-α) or even lower (IFN-γ, IL-1β and IL-6) (Figure 4). These results suggest that the coimmunization strategy could induce anti-inflammatory effects *in vivo* by downregulating the expression of IFN-γ and TNF-α. We checked whether CD4^+^ T cells had infiltrated into brain tissues. Immunohistochemistry revealed that there were small numbers of CD4^+^ T cells in the brains of the protein-vaccinated group, indicating that the source of increased inflammation was infiltrated T cells (Additional file 2: Figure S3).

### Coimmunization suppresses T-cell proliferation by inducing CD25^−^ iTreg cells

**Figure 5:**
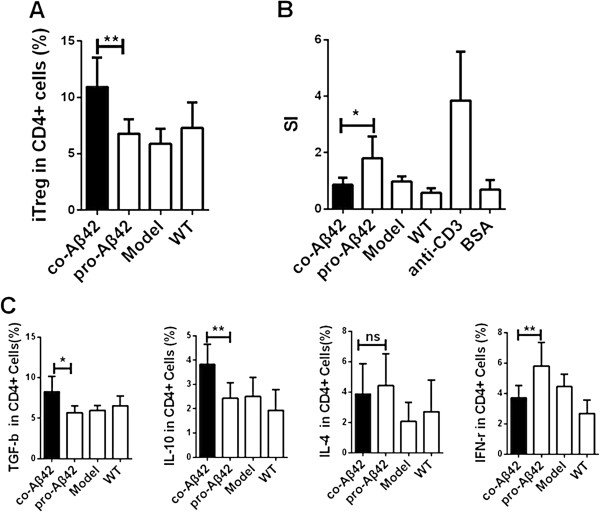
**Coimmunization suppresses T-cell proliferation by inducing CD25**^**− **^**antigen-specific regulatory T cells.** Splenocytes were isolated from vaccinated and control mice 7 days after the fifth immunization and used for intracellular staining or for proliferation assays (*n* = 6). **(A)** Frequency of FoxP3^+^ cells in the total CD4^+^ T cells after the coimmunization compared with other groups by performing fluorescence-activated cell sorting analysis (FACS) analysis. **(B)** The level of T-cell proliferation was measured by 3-(4,5-dimethylthiazol-2-yl)-2,5-diphenyltetrazolium bromide assay. The splenocytes were restimulated for 3 days *in vitro* using amyloid-β42 (Aβ42) protein as a specific antigen, bovine serum albumin (BSA) as a nonspecific antigen or anti-CD3 as a positive control. We added anti-CD28 antibody to all T cells as a costimulant. The cell proliferation rate is expressed as the stimulation index (SI). **(C)** Splenocytes were restimulated for 12 hours *in vitro* using Aβ42 protein as a specific antigen and blocked with brefeldin A for 8 hours. The lymphocytes were first gated on CD4^+^ T cells and then used for intracellular staining to detect transforming growth factor β (TGF-β) and interleukin 10 (IL-10), IL-4 and interferon γ (IFN-γ) followed by FACS analysis. A result typical of three independent experiments is shown. All data are presented as mean ± SD. Statistical analysis was performed using parametric one-way analysis of variance, and *t*-tests were used to compare two groups. *P < 0.05, **P < 0.01 compared with protein-vaccinated model mice. co-Aβ42, APP_695_ mice immunized with co-amyloid-β42 vaccine; ns, Not significant; pro-Aβ42, APP_695_ mice immunized with amyloid-β42 protein vaccine; WT, Wild type.

We previously demonstrated that coimmunization induced high titers of IgG against protein antigen. Coimmunization also induced antigen-specific iTreg cells to suppress CD4 T-cell responses in an antigen-specific manner [33]. To test whether this would also hold true for anti-Aβ42 coimmunization, we performed a T-cell proliferation assay 7 days after the fifth immunization (after the behavior tests had been completed). As expected, Aβ42 protein immunization induced a twofold stimulation compared to immunized control mice, whereas coimmunization did not induce any T-cell proliferation (Figure 5A). The suppression of T-cell responses was apparently associated with increases in CD25^−^ iTreg cell levels in the coimmunized mice (Figure 5B) in a dose-dependent manner (Additional file 1: Figure S1).

We next tested whether the iTreg cells affected T-cell production of inflammatory cytokines. After immunization, CD4^+^ T cells were isolated, and intracellular staining was performed. As shown in Figure 5C, the level of IFN-γ was downregulated, but levels of Foxp3, IL-10 and TGF-β were upregulated, by the coimmunization regimen compared to the Aβ42 protein–immunized group. In contrast, no effect on the level of IL-4 was observed (Figure 5C). These results suggest that the coimmunization protocol induced a Th1-suppressive response, but did not affect the humoral response.

### Coimmunization prevents induced experimental autoimmune encephalomyelitis

**Figure 6:**
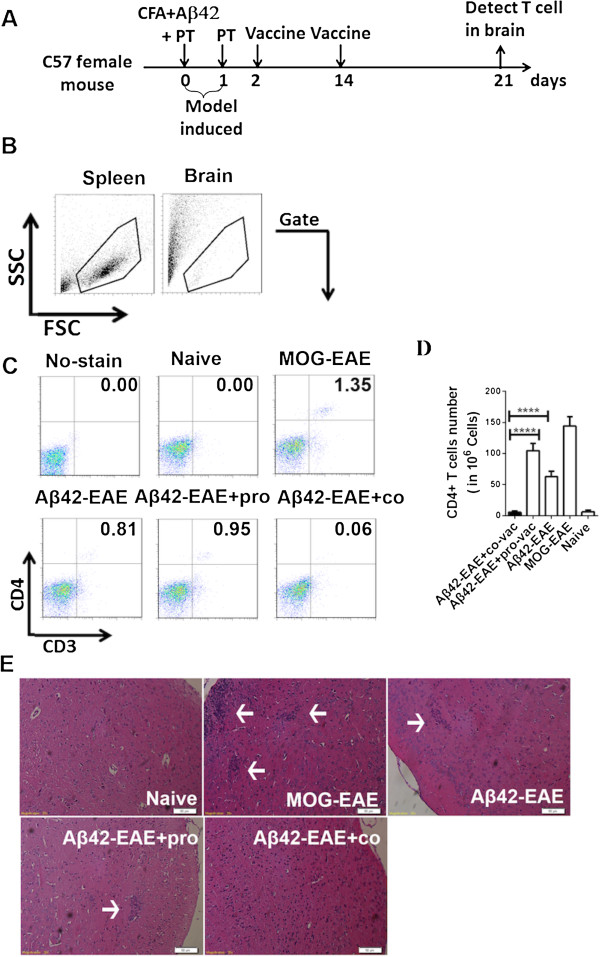
**Coimmunization prevents autoimmune encephalomyelitis induced by amyloid-β42 protein. (A)** Schematic representation of amyloid-β42 autoimmune encephalomyelitis (Aβ42-EAE) induction and vaccination. EAE was induced by immunization with Aβ42 protein + complete Freund’s adjuvant (CFA) + pertussis toxin (PT) (Aβ42-EAE model) on day 0, and then, on day 1, PT was injected a second time. The EAE-induced mice were further coimmunized or protein-immunized on days 2 and 14. T cells infiltrating the brain were assessed by isolating T cells from the brain on day 21. **(B)** Single-cell suspensions of brain cells taken from EAE-induced animals were isolated with or without vaccination or from naïve C57BL/6 mice and then stained on day 21. FSC, Forward scatter; SSC, Side scatter. **(C)** The cells were immunostained for CD3 and CD4 and analyzed by fluorescence-activated cell sorting (FACS). The upper left panel shows cells from naïve mice with no antibody staining, followed by antibody-stained cells from the naïve mice, from the myelin oligodendrocyte glycoprotein (MOG)–induced EAE (MOG-EAE) model, from the Aβ42-induced EAE model, from the Aβ42-induced EAE mice vaccinated with Aβ42 protein vaccine or from the coimmunized mice. **(D)** Graph showing the number of CD4^+^ T cells in brain. The number of double-positive CD3^+^ and CD4^+^ T cells per 10^6^ brain cells from the groups of six animals represented in **(C)** were statistically analyzed and plotted. **(E)** Histopothological examination of brain cortex on the sixth day after EAE model induction, stained with hematoxylin and eosin. The naïve tissue section was taken from naïve mice without any treatment or induction. The results shown are representative of three independent experiments. Statistical analysis was performed using parametric one-way analysis of variance, and *t*-tests were used to compare two groups. The arrows point to the lymphocytes areas. The bars represent 50 micrometer in length. co, Coimmunization; pro, Protein immunization. All data are presented as mean ± SD. ****P < 0.0001 compared with protein-vaccinated Aβ42-EAE mice.

Because vaccinations may cause brain inflammation that leads to meningoencephalitis, minimizing the risk of such inflammation and meningoencephalitis has to be a major goal in the development of an AD vaccine. EAE represents an excellent model for studying T-cell-mediated autoimmune disease in the brain and mimics Aβ42 vaccine–induced meningoencephalitis [48]. To test the efficacy of the coimmunization strategy in preventing EAE, we first established an EAE animal model by immunizing mice on day 0 with either MOG35–55 peptide + CFA + PT (MOG-EAE model) or with Aβ42 protein + CFA + PT (Aβ42-EAE model) and then with PT on day 1. We found that the pVAX1-Aβ42 plasmid was expressed by day 2 after transfection (Additional file 3: Figure S4). The coimmunization vaccine can induce the signaling pathway in dendritic cells at this time point [34,49], and the DNA plasmid vaccine can induce T-cell responses from day 3 after immunization [50]. Therefore, the EAE-induced mice were further coimmunized or protein-immunized on days 2 and 14. A schematic of the strategy is depicted in Figure 6A. T cells infiltrating the brain were isolated and analyzed by flow cytometry on day 21 after the single-cell suspensions were stained with anti-CD3 and anti-CD4 antibodies (Figure 6C). A large number of CD3^+^ and CD4^+^ T cells had infiltrated into the brains of MOG-induced or Aβ42-induced EAE mice, possibly causing meningoencephalitis. Even larger brain infiltrations were observed in the Aβ42-induced EAE mice after subsequent Aβ42 protein immunization (Figures 6C and 6D). However, in sharp contrast, the number of T cells was dramatically reduced to the level in the naïve group when the mice were coimmunized (*P* < 0.0001) (Figures 6C and 6D). These results were confirmed in histopathological analysis of H&E-stained brain sections. MOG-EAE and Aβ42-EAE caused large clusters of infiltrated T cells (Figure 6E), whereas no T-cell infiltration was detected in the coimmunized animals, thus resembling the situation in naïve mice (Figure 6E). This finding result demonstrates that coimmunization with Aβ42 DNA and Aβ42 protein induced a protective response against brain inflammation.

### Coimmunization-induced regulatory T cells suppressed brain inflammation

**Figure 7:**
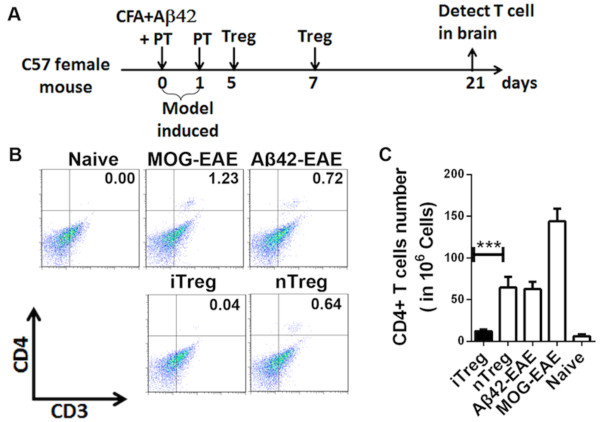
**Effects of induced antigen-specific regulatory T cell transfer on T-cell infiltration in the brain tissue in the amyloid-β42-induced autoimmune encephalomyelitis model.** CD4^+^CD25^−^Foxp3^+^ induced antigen-specific regulatory T cells (iTregs) were isolated from coimmunized Foxp3 enhanced green fluorescent protein (Foxp3-eGFP) mice on day 7 after the second coimmunization and adoptively transferred into amyloid-42β-induced autoimmune encephalomyelitis (Aβ42-EAE) model mice on days 5 and 12 after EAE induction. **(A)** Schematic representation of iTreg cell transfer to prevent Aβ42-EAE. iTregs were isolated from Foxp3-eGFP mice after the second coimmunization and adoptively transferred into Aβ42-EAE model mice on days 5 and 12 after the model was induced. CD4^+^CD25^+^Foxp3^+^nTreg transfers were used as a control. CFA, Complete Freund’s adjuvant; nTreg, näive regulatory T cell; PT, Pertussis toxin. **(B)** Single-cell suspensions of brains were isolated from EAE-induced animals with or without vaccinations, or from naïve C57BL/6 mice, and stained on day 21. The cells were immunostained for CD3 and CD4 and analyzed by fluorescence-activated cell sorting. MOG, Myelin oligodendrocyte glycoprotein. **(C)** The number of double-positive CD3^+^ and CD4^+^ T cells per 10^6^ brain cells taken from the groups of six animals detected the same way as in **(B)** were statistically analyzed and plotted. The results shown are typical of three independent experiments. All data are presented as mean ± SD. Statistical analysis was performed using parametric one-way analysis of variance, and *t*-tests were used to compare two groups. ***P < 0.001 compared with Aβ42-EAE mice.

We wanted to examine whether the iTreg cells acted as the main suppressors of Aβ42 antigen-specific T cells. To this end, we isolated iTreg cells from coimmunized Foxp3-eGFP mice and adoptively transferred them into Aβ42-induced EAE model animals as recipients on days 5 and 12 after EAE induction (Figure 7A). As a control, näive regulatory T cells (nTregs) were transferred. T cells isolated from each brain were analyzed by FACS (Figure 7B), and the sum of results is presented as a bar graph in Figure 7C. On average, more than 100 T cells per 10^6^ brain cells infiltrated brains of MOG-induced EAE mice, and about 70 T cells per 10^6^ brain cells infiltrated Aβ42-induced EAE mice. Transferring nTregs had no impact on T-cell infiltration, but transfer of iTregs significantly reduced T-cell infiltration in the brains of Aβ42-induced EAE mice (*P* < 0.001) (Figure 7C). This result demonstrates that the iTreg cells induced by coimmunization did indeed suppress Teff cell infiltration into the brain.

These data demonstrate that the coimmunization regimens with Aβ42 protein and Aβ42 coding DNA-induced iTregs strongly reduced brain inflammation and infiltration of T cells into the brain. At the same time, a robust anti-Aβ42 antibody response was elicited that effectively reduced plaque formation. Therefore, coimmunization is an attractive and novel strategy for use in AD vaccine development.

## Discussion

In this study, the co-immunization strategy that we developed was applied for the first time in an AD mouse model. Our goal in mixing the Aβ42 protein and Aβ42 DNA vaccine was to achieve high levels of anti-Aβ42 antibodies with concurrent inhibition of T-cell-mediated inflammation. This should ameliorate cognitive deficits while lowering the adverse effects of neuroinflammation in conventionally vaccinated mice and humans. We demonstrate that the coimmunization of DNA encoding Aβ42 plus Aβ42 protein in APP_695_ mice indeed induced higher levels of IgG against Aβ, resulting in reduction of Aβ load and dramatically improved behavior in Morris water maze and open-field tests. The coimmunization induced high levels of iTreg with a strong ability to suppress CD4^+^ T-mediated inflammation. This resulted not only in suppression of T-cell proliferation but also, importantly, in reduced expression of inflammatory cytokines, including IFN-γ, IL-1β, IL-6 and TNF-α in brain tissue samples. We found small numbers of CD4^+^ T cells in the brains taken from the protein vaccine group (Additional file 2: Figure S3). So we speculated that the source of increased inflammatory stimuli, such as IFN-γ and TNF-α, was the infiltrated T cells. Expression of these cytokines is thought to lead to T cell infiltration and is associated with in situ lesions [5,51,52]. We also looked for microhemorrhaging in the brain tissue by applying Prussian blue staining and found that this coimmunization vaccine did not induce microhemorrhaging (Additional file 4: Figure S2). We did find one mouse in the protein-immunized group that had a Prussian blue–positive spot, but we could not find cerebral amyloid angiopathy (CAA) when we applied Congo red staining in both immunized groups up to 10 months after their initial immunization, suggesting that these animals may not yet have reached the age of intense CAA. Therefore, future studies in older mice are warranted to investigate this problem.

The protein Aβ42 that we have used in this study has one main difference from AN-1792, the vaccine used in a phase II trial conducted with human patients [10]. We used CFA and IFA as adjuvants in our protein vaccine, whereas QS-21 was the adjuvant used in AN-1792 [11]. Both adjuvants not only augment antibody responses but also elicit strong helper T-cell responses, producing cytokines such as IFN-γ [53-58], which can cause perivenular inflammatory encephalomyelitis during vaccination [5]. In earlier animal experiments, however, CFA and IFA were demonstrated to improve the efficiency of Aβ42 antibody production to attenuate AD-like pathology in the PDAPP mouse [8].

Our data show that the efficacy of the coimmunization vaccine to reduce plaque load is comparable to a “conventional”, well-established protein vaccine, but at the same time is superior because it causes less side effects.

The main concern with regard to current protein-based Aβ42 AD vaccines is that they can cause meningoencephalitis in AD patients, because vaccination with Aβ42-based vaccines might induce T-cell activation and infiltration into the central nervous system [10]. To avoid this problem, several alternative approaches are currently being developed, including the use of monoclonal antibody against Aβ42 for passive immunization or a truncated form of Aβ42 as a vaccine, such as Aβ1−15-DT conjugate, which could increase antibody titers by adding nonrelevant epitopes of a DT carrier for T helper cells [14]. Researchers who have used these approaches have demonstrated that Aβ deposits in AD mouse models were effectively cleared, and several of these approaches were subsequently tested in clinical settings [28,59-61]. The aim of all of these approaches is to prevent the activation of CD4^+^ T-cell responses caused by the T-cell epitope located in the Aβ15-42 fragment. However, to date, no published clinical trial data or other evidence has shown that these approaches can be used to develop an effective vaccine against AD and prevent encephalitis or other adverse effects.

iTreg cells regulate T-cell-mediated immune reactions, thereby controlling autonomous immune responses. The use of Treg cells to treat inflammatory reactions has been considered a promising strategy, particularly against autoimmune diseases, allergies and asthma [62-65]. Although researchers have demonstrated powerful suppressive effects of iTreg against Teff cells in numerous studies, no strategy designed to develop an AD vaccine using iTregs to prevent T-cell-mediated brain inflammation has been tested. In our present study, we show for the first time that coimmunization of Aβ42 can indeed inhibit T cells by inducing iTreg cells in normal C57BL/6 mice (Additional file 1: Figure S1) and in APP_695_ mice (Figure 5).

To establish a link between iTreg cells and reduction of T-cell-mediated inflammation, we used an Aβ42-induced EAE model. The aim was to mimic the CD4^+^ T-cell infiltration into the brain that was observed with the use of AN-1792. We observed that this coimmunization significantly inhibited the formation of encephalitis by inducing a group of iTregs to inhibit the migration of CD4^+^ T cells into the brain. To conclusively demonstrate that the iTreg cells induced by coimmunization suppressed T-cell-mediated infiltration and inflammation in brain tissue, we adoptively transferred iTreg cells obtained from a donor mouse that had been coimmunized by the regimens into EAE-induced mice with inflammatory brain tissues. The results show that iTreg cells could indeed inhibit inflammatory CD4^+^ T-cell infiltration and thus reduce the number of T cells in brain tissue. Altogether, these results demonstrate that coimmunization inhibited the inflammatory response that was induced by the protein vaccine and that the inhibition was produced by induction of high levels of iTregs. The iTregs strongly suppressed CD4^+^ T-cell proliferation, inflammatory cytokine expression and migration into the inflammation site.

## Conclusions

The data derived from our study of the APP_695_ mice described in this article demonstrate that the coimmunization regimens with Aβ42 protein and Aβ42-coding DNA induced both high titers of antibody against Aβ42, which effectively reduced plaque formation in this AD mouse model, and induced iTregs that strongly reduced brain inflammation and infiltration of T cells into the brain. AD development was ameliorated. Therefore, the results are a proof of concept and indicate that the coimmunization strategy reported herein should be further developed with the goal of testing it in human patients.

## Abbreviations

AD: Alzheimer’s disease; APC: Allophycocyanin; CBA: Cytometric bead array; EAE: Experimental autoimmune encephalomyelitis; eGFP: Enhanced green fluorescent protein; ELISA: Enzyme-linked immunosorbent assay; iTreg: Induction of antigen-specific regulatory T cell; nTreg: Naïve regulatory cell; PBS: Phosphate-buffered saline; Teff: T-effector cell.

## Competing interests

A patent application has been filed based on the content of this manuscript.

## Authors' contributions

SW, BWa, YY and SG conceived of and designed the experiments. SW, YY, DW, LZ, XX, BWu, CL, HX, XL and YH performed the experiments. SW, YY, SG, LZ and BWa analyzed the data. CK and LZ contributed the reagents, materials and analysis tools. SW, BWa and CK wrote the manuscript. All authors read and approved the final manuscript.

## Supplementary Material

Additional file 1: Figure S1 Effects of coimmunization on immune responses**.** Groups of C57BL/6 mice (*n* = 6) were immunized by coimmunization with protein/DNA at various ratios (100 μg/100 μg, 100 μg/200 μg, 100 μg/300 μg and 200 μg/100 μg), protein vaccine alone or DNA vaccine alone, or they were un-vaccinated as controls. **(A)** Total anti-Aβ IgG was analyzed by ELISA on day 7 after the third immunization. Mean titers are expressed as log_10_ values. **(B)** Splenocytes were isolated from each group on day 7 after the third immunization and stimulated for 3 days *in vitro* using Aβ42 protein as the specific antigen, BSA as a nonspecific antigen or anti-CD3 as a positive control stimulant. All of the T cells were given anti-CD28 as a costimulant. The level of T-cell proliferation was evaluated by MTT assay, and the data are expressed as a stimulation index. **(C)** The number of CD4^+^CD25^−^Foxp3^+^ iTreg cells as a percentage of total CD4^+^ T cells was analyzed by FACS analysis. Data shown are the results of three independent experiments. All data are presented as mean ± SD. Statistical analysis was performed using parametric one-way ANOVA, and *t*-tests were used for comparing two groups. *P < 0.05, **P < 0.01 compared with Aβ42 protein vaccinated mice. Click here for file

Additional file 2: Figure S3 CD4^+^ cells staining of brain sections from AD model mice after immunization. Brain sections were taken from 14-month-old AD mice (*n* = 5) after the fifth immunization and stained for immunohistochemistry analysis. The sections were incubated with anti-CD4 as primary antibody. WT refers to littermate negative controls, and the model used was APP_695_ mice without immunization. Co-Aβ42 refers to APP_695_ mice immunized with co-Aβ42 vaccine, and pro-Aβ42 refers to APP_695_ mice immunized with protein vaccine. Cortical sections were viewed under a microscope with visible light. Click here for file

Additional file 3: Figure S4 Expression of pVAX1-Aβ42 plasmid transfected into BHK-21 cells. The pVAX1-Aβ42 plasmid was transfected into BHK-21 cells with Lipofectamine 2000 reagent. The cells were collected at 48 hours after transfection. The expression of the plasmid was detected by Western blot analysis, a commercially purchased Aβ42 peptide was used as a positive control and BHK-21 cells without transfection were used as a negative control. The positive Aβ42 peptide was about 4 kDa, and GAPDH was about 30 to 40 kDa. Data shown in each panel are derived from one of two independent experiments with similar results. Click here for file

Additional file 4: Figure S2 Prussian blue staining of brain sections from AD model mice after immunization. For microhemorrhage staining, brain sections from 14-month-old AD model mice (*n* = 5) after the fifth immunization were fixed and stained using the Prussian blue method. WT means littermate negative controls, model was APP_695_ mice without immunization. Co-Aβ42 refers to the APP_695_ mice immunized with co-Aβ42 vaccine, and pro-Aβ42 refers to the APP_695_ mice immunized with protein vaccine. Cortical sections were viewed under a microscope with visible light. Click here for file
