# Supplementary figures and images for: A coimmunization vaccine of Aβ42 ameliorates cognitive deficits without brain inflammation in an Alzheimer’s disease model
Source: Alzheimers Res Ther. 2014 May 12;6(3):26. doi: 10.1186/alzrt256 (PMC4075150; doi:10.1186/alzrt256)

## Slide 1
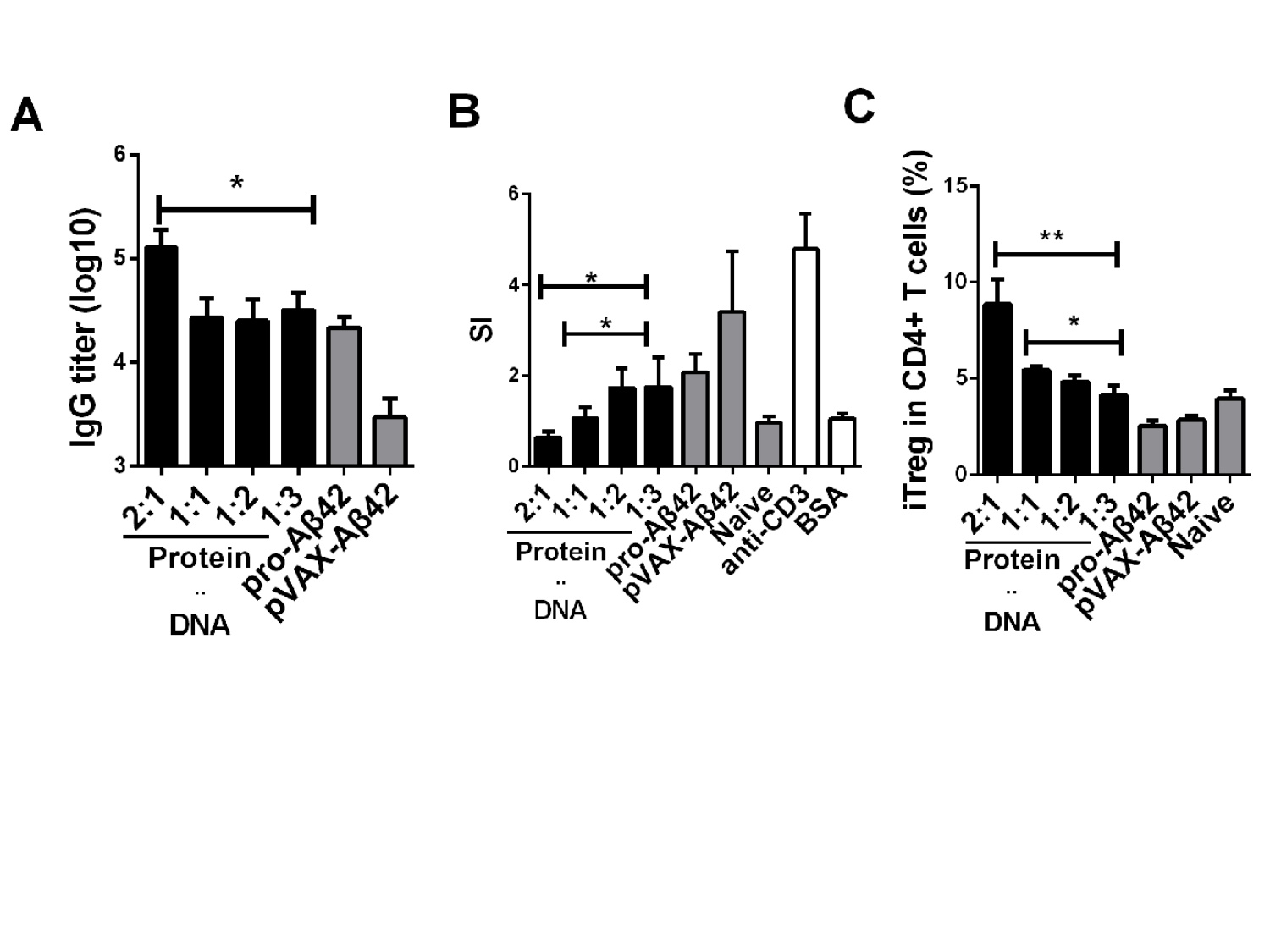

Supplement: Additional file 1: Figure S1 — Effects of coimmunization on immune responses. Groups of C57BL/6 mice (n = 6) were immunized by coimmunization with protein/DNA at various ratios (100 μg/100 μg, 100 μg/200 μg, 100 μg/300 μg and 200 μg/100 μg), protein vaccine alone or DNA vaccine alone, or they were un-vaccinated as controls. (A) Total anti-Aβ IgG was analyzed by ELISA on day 7 after the third immunization. Mean titers are expressed as log10 values. (B) Splenocytes were isolated from each group on day 7 after the third immunization and stimulated for 3 days in vitro using Aβ42 protein as the specific antigen, BSA as a nonspecific antigen or anti-CD3 as a positive control stimulant. All of the T cells were given anti-CD28 as a costimulant. The level of T-cell proliferation was evaluated by MTT assay, and the data are expressed as a stimulation index. (C) The number of CD4+CD25−Foxp3+ iTreg cells as a percentage of total CD4+ T cells was analyzed by FACS analysis. Data shown are the results of three independent experiments. All data are presented as mean ± SD. Statistical analysis was performed using parametric one-way ANOVA, and t-tests were used for comparing two groups. *P < 0.05, **P < 0.01 compared with Aβ42 protein vaccinated mice. [file alzrt256-S1.pptx]

## Slide 1
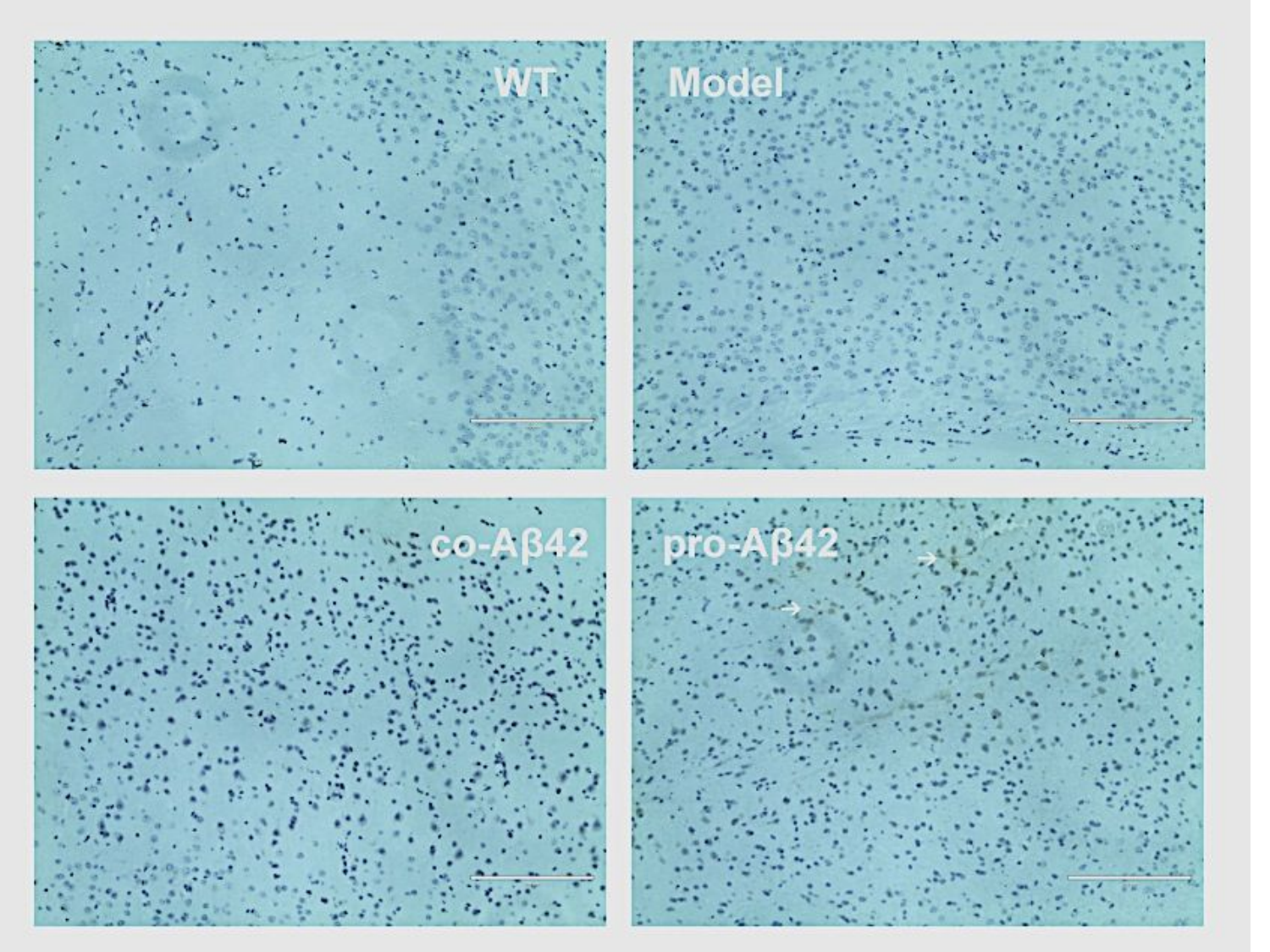

Supplement: Additional file 2: Figure S3 — CD4+ cells staining of brain sections from AD model mice after immunization. Brain sections were taken from 14-month-old AD mice (n = 5) after the fifth immunization and stained for immunohistochemistry analysis. The sections were incubated with anti-CD4 as primary antibody. WT refers to littermate negative controls, and the model used was APP695 mice without immunization. Co-Aβ42 refers to APP695 mice immunized with co-Aβ42 vaccine, and pro-Aβ42 refers to APP695 mice immunized with protein vaccine. Cortical sections were viewed under a microscope with visible light. [file alzrt256-S2.pptx]

## Slide 1
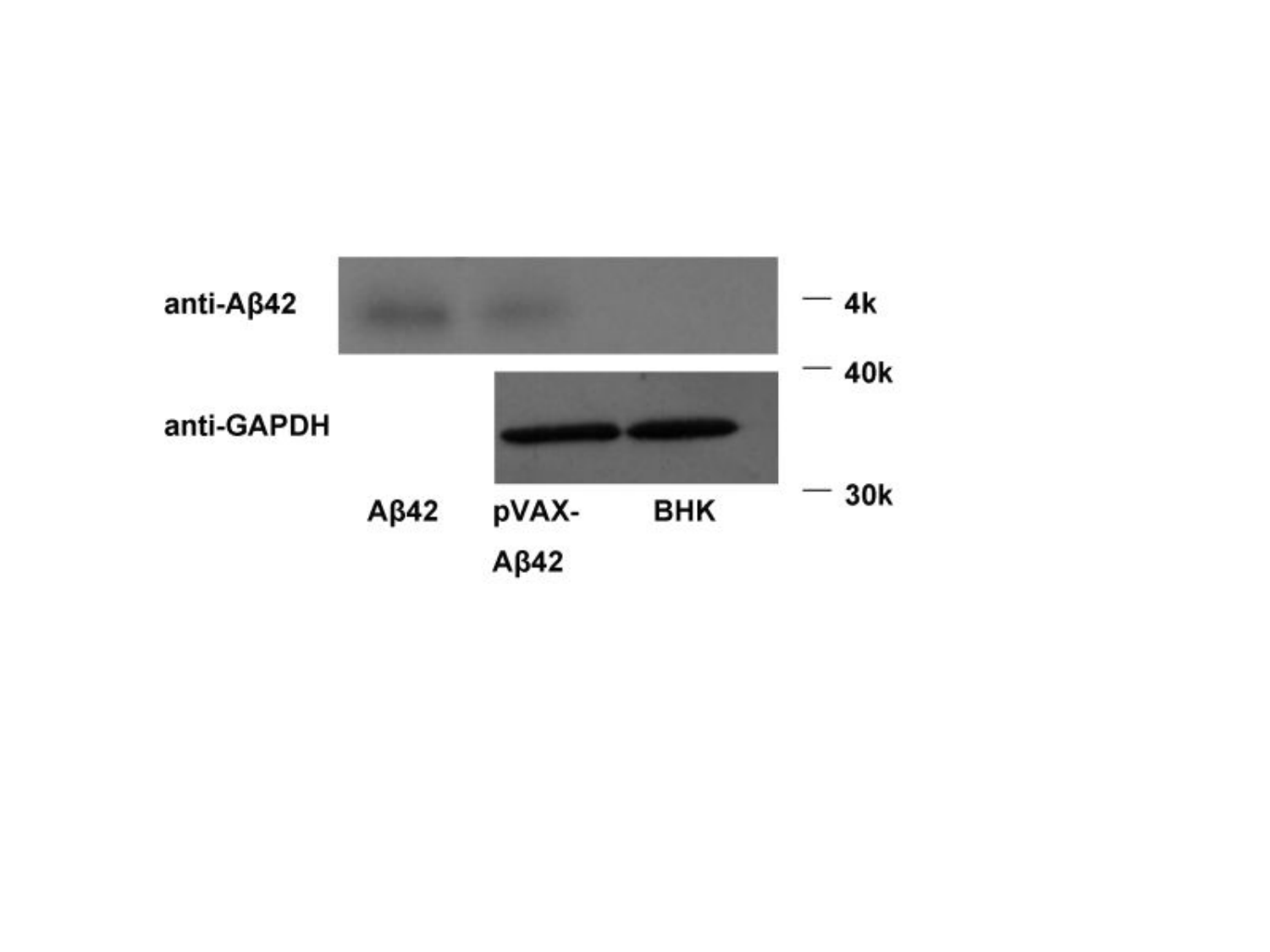

Supplement: Additional file 3: Figure S4 — Expression of pVAX1-Aβ42 plasmid transfected into BHK-21 cells. The pVAX1-Aβ42 plasmid was transfected into BHK-21 cells with Lipofectamine 2000 reagent. The cells were collected at 48 hours after transfection. The expression of the plasmid was detected by Western blot analysis, a commercially purchased Aβ42 peptide was used as a positive control and BHK-21 cells without transfection were used as a negative control. The positive Aβ42 peptide was about 4 kDa, and GAPDH was about 30 to 40 kDa. Data shown in each panel are derived from one of two independent experiments with similar results. [file alzrt256-S3.pptx]

## Slide 1
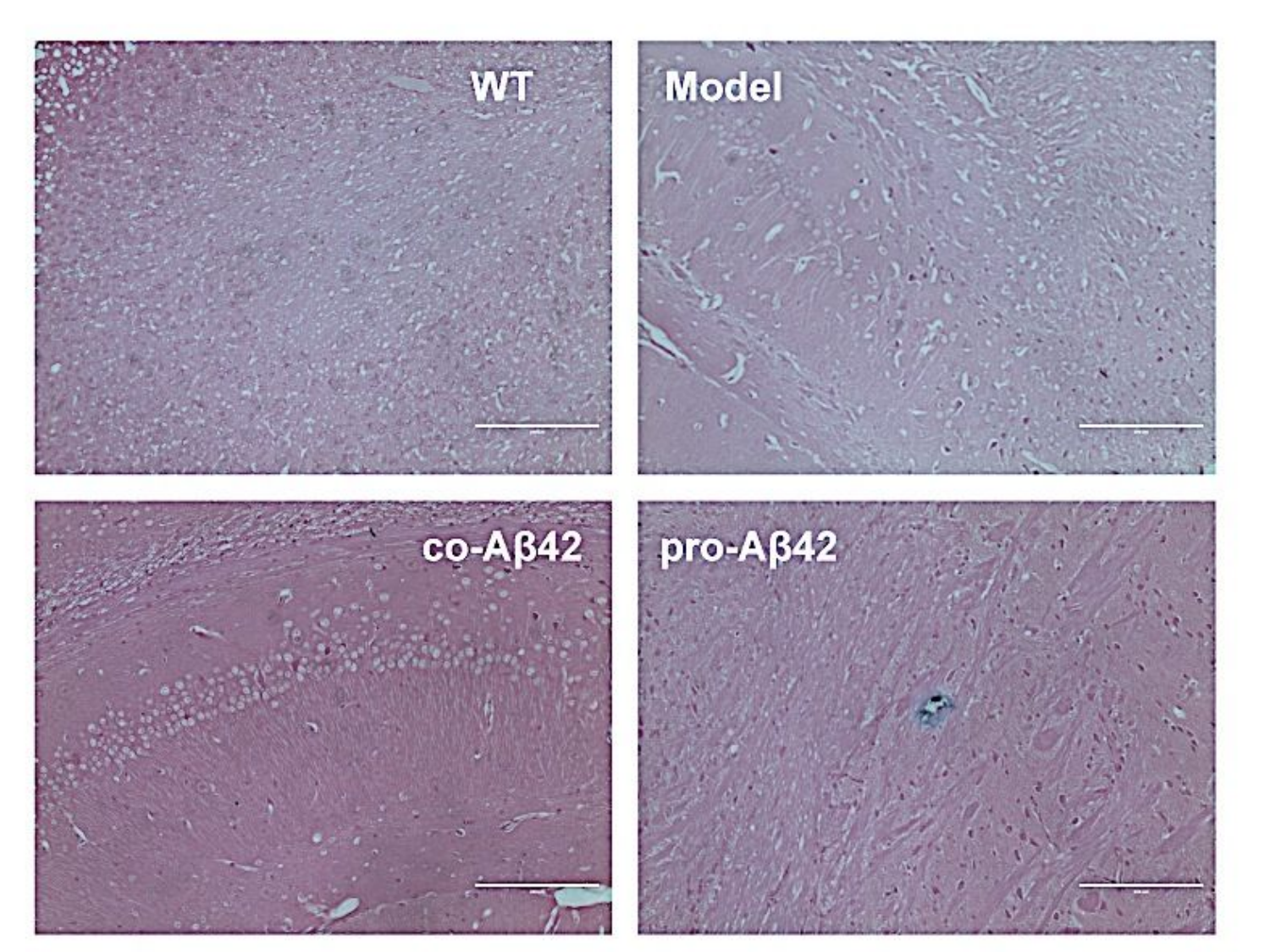

Supplement: Additional file 4: Figure S2 — Prussian blue staining of brain sections from AD model mice after immunization. For microhemorrhage staining, brain sections from 14-month-old AD model mice (n = 5) after the fifth immunization were fixed and stained using the Prussian blue method. WT means littermate negative controls, model was APP695 mice without immunization. Co-Aβ42 refers to the APP695 mice immunized with co-Aβ42 vaccine, and pro-Aβ42 refers to the APP695 mice immunized with protein vaccine. Cortical sections were viewed under a microscope with visible light. [file alzrt256-S4.pptx]
